# Targeted Induction of Endogenous VDUP1 by Small Activating RNA Inhibits the Growth of Lung Cancer Cells

**DOI:** 10.3390/ijms23147743

**Published:** 2022-07-13

**Authors:** Ki Hwan Park, Jeong-Wook Yang, Joo-Hee Kwon, Hyunju Lee, Yeo Dae Yoon, Byeong Jo Choi, Myeong Youl Lee, Chang Woo Lee, Sang-Bae Han, Jong Soon Kang

**Affiliations:** 1 Laboratory Animal Resource Center, Korea Research Institute of Bioscience and Biotechnology, 30 Yeongudanji, Cheongwon, Cheongju 28116, Chungbuk, Korea; brightnessd@nate.com (K.H.P.); z7v8@kribb.re.kr (J.-W.Y.); juhee@kribb.re.kr (J.-H.K.); hyunju35@kribb.re.kr (H.L.); yunyd76@kribb.re.kr (Y.D.Y.); byung127@kribb.re.kr (B.J.C.); myong@kribb.re.kr (M.Y.L.); changwoo@kribb.re.kr (C.W.L.); 2 College of Pharmacy, Chungbuk National University, 194-21, Osongsaengmyung-1, Heungdeok, Cheongwon, Cheongju 28116, Chungbuk, Korea; shan@chungbuk.ac.kr

**Keywords:** RNAa, saRNA, VDUP1, histone modification, Ago2, lung cancer

## Abstract

Recent studies have reported that small double-strand RNAs (dsRNAs) can activate endogenous genes via an RNA-based promoter targeting mechanism termed RNA activation (RNAa). In the present study, we showed that dsVDUP1-834, a novel small activating RNA (saRNA) targeting promoter of vitamin D_3_ up-regulated protein 1 (VDUP1) gene, up-regulated expression of VDUP1 at both mRNA and protein levels in A549 lung cancer cells. We also demonstrated that dsVDUP1-834 inhibited cell proliferation in A549 lung cancer cells. Further studies showed that dsVDUP1-834 induced cell-cycle arrest by increasing p27 and p53 and decreasing cyclin A and cyclin B1. In addition, knockdown of VDUP1 abrogated dsVDUP1-834-induced up-regulation of VDUP1 gene expression and related effects. The activation of VDUP1 by dsVDUP1-834 was accompanied by an increase in dimethylation of histone 3 at lysine 4 (H3K4me2) and acetylation of histone 3 (H3ac) and a decrease in dimethylation of histone 3 at lysine 9 (H3K9me2) at the target site of VDUP1 promoter. Moreover, the enrichment of Ago2 was detected at the dsVDUP1-834 target site, and Ago2 knockdown significantly suppressed dsVDUP1-834-mediated inhibition of cell proliferation and modulation of cell-cycle regulators. Taken together, the results presented in this report demonstrate that dsVDUP1-834 induces VDUP1 gene expression by epigenetic changes, resulting in cell growth inhibition and cell-cycle arrest. Our results suggest that targeted induction of VDUP1 by dsVDUP1-834 might be a promising therapeutic strategy for the treatment of lung cancer.

## 1. Introduction

Small double-strand RNA (dsRNA) molecules, such as a small interfering RNA (siRNA) or microRNA (miRNA), were initially shown to trigger RNA interference (RNAi), a mechanism of gene silencing, which leads to the degradation of target mRNA and subsequent post-transcriptional gene silencing [1]. However, it has been reported that dsRNAs can also induce sequence-specific transcriptional gene activation by targeting gene promoter regions [2,3]. This phenomenon was termed RNA-induced gene activation or RNAa, and the gene-activating dsRNA was termed a small activating RNA (saRNA) [2]. Epigenetic modification, including demethylation and acetylation of histones, is the main mechanism of gene activation in RNAa. RNAa was known to depend on Argonaute (Ago) proteins, such as Ago2, and exhibit unique kinetics characterized by prolonged (10~14 days) induction of gene expression, which is different from the kinetics of siRNA-mediated gene silencing [4].

Vitamin D_3_ up-regulated protein 1 (VDUP1), also known as thioredoxin-interacting protein (TXNIP) or thioredoxin-binding protein 2 (TBP-2), was originally characterized as an up-regulated gene in HL-60 cells treated with 1,25-dihydrovitamin D_3_ [5]. Further study demonstrated that VDUP1 interacts with thioredoxin and acts as a negative regulator of thioredoxin function and expression [6]. In particular, VDUP1 was suggested as a tumor-suppressor gene, which was commonly reduced in many cancers by various epigenetic and genetic mechanisms [7]. Several groups also reported that VDUP1 exerts an anti-cancer role in rodent models of carcinogenesis, including hepatocarcinogensis [8], bladder carcinogenesis [9], and gastric carcinogenesis [10]. In addition, it has been reported that VDUP1 expression is down-regulated in lung cancer tissues [11]. Moreover, sodium butyrate, a well-known histone deacetylase (HDAC) inhibitor, exerted an anti-cancer effect against human lung cancer cells by induction of the VDUP1 gene [12].

To investigate whether specific induction of VDUP1 gene expression can be a promising strategy for anti-cancer treatment, we screened and selected a novel saRNA, dsVDUP1-834, which specifically targets human VDUP1 promoter and up-regulates VDUP1 gene expression. In this study, we examined the effect of dsVDUP1-834 on cell proliferation, cell-cycle distribution, and the expression of cell-cycle regulators in A549 lung cancer cells. We also examined epigenetic changes induced by dsVDUP1-834 and the involvement of Ago2 in dsVDUP1-834-mediated effects. This study suggests that a specific induction of VDUP1 by saRNA might be a promising therapeutic strategy for the treatment of lung cancer.

## 2. Results

### 2.1. Induction of VDUP1 Expression by dsVDUP1-834 in A549 Cells

**Figure 1:**
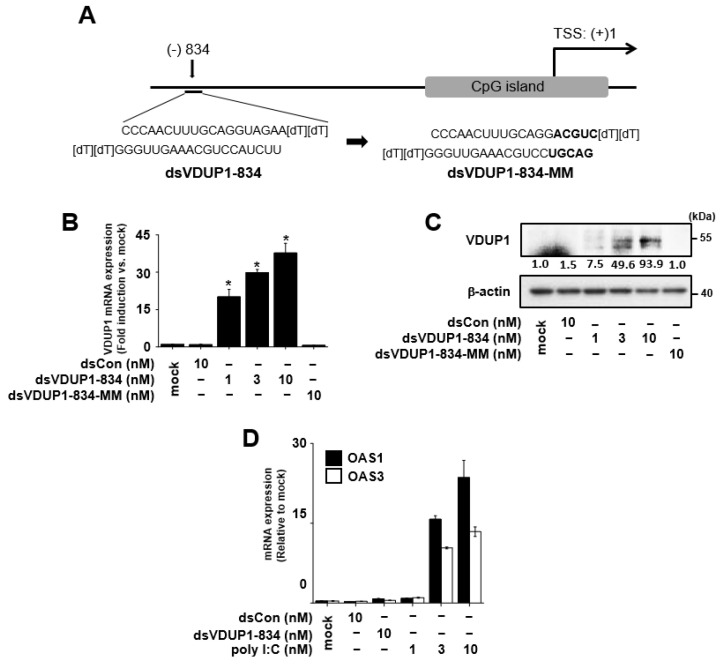
dsVDUP1-834 transfection induces VDUP1 expression in a dose-dependent manner in A549 cells. (**A**) Schematic representation of VDUP1 promoter showing target site and sequences of saRNAs. A549 cells were transfected with dsCon or indicated concentrations of saRNAs for 96 h. (**B**) The mRNA expression of VDUP1 was determined by qRT-PCR. (**C**) The protein expression of VDUP1 was assessed by Western immunoblot analysis. Normalized relative intensities are shown below the bands. (**D**) OAS1 and OAS3 mRNA expression was analyzed by qRT-PCR. Data are represented as mean ± S.D of triplicate determinations. * denotes that the response is significantly different from mock-treated group as determined by Dunnett’s *t*-test at *p* < 0.05.

We screened various saRNAs and selected dsVDUP1-834, which was designed to target VDUP1 promoter at positions -834 relative to the transcription start site, as a candidate saRNA for induction of VDUP1 expression (Figure 1A). A control saRNA (dsCon) lacking homology to any human sequences was also designed as described previously [2] and used as a negative control for all experiments. A549 cells were selected as a model cell line because the expression of VDUP1 was suppressed, and the magnitude of VDUP1 induction by dsVDUP1-834 was dramatic compared to other cancer cell lines (Supplementary Figure S1).

To investigate the effect of dsVDUP1-834 on VDUP1 expression, we transfected dsVDUP1-834 (at 1, 3, and 10 nM) into A549 cells and examined VDUP1 mRNA and protein expression. As shown in Figure 1B,C and Supplementary Figure S2, VDUP1 mRNA and protein expression were induced by dsVDUP1 in a concentration-dependent manner. To exclude the possibility that VDUP1 activation has resulted from an off-target effect, we generated a mutated dsVDUP1-834 (dsVDUP1-834-MM) by changing the last five bases (relative to 5′ end of the anti-sense strand) of dsVDUP1-834 (Figure 1A) and investigated its effect on VDUP1 expression. Figure 1B,C and Supplementary Figure S2 show that dsVDUP1-834-MM did not cause induction of VDUP1 expression.

In addition, to determine whether dsVDUP1-834 transfection induces non-specific interferon (IFN) responses, we analyzed OAS1 (2′,5′-oligoadenylate synthase 1) and OAS3 (2′,5′-oligoadenylate synthase 3), two classic interferon response genes, by qRT-PCR. As shown in Figure 1D, dsVDUP1-834 did not affect the mRNA expression of OAS1 and OAS3, whereas poly I:C (polyinosinic-polycytidylic acid) dose-dependently increased the expression of these genes (Figure 1D).

### 2.2. dsVDUP1-834 Inhibits Cell Growth and Induces Cell-Cycle Arrest in A549 Cells

**Figure 2:**
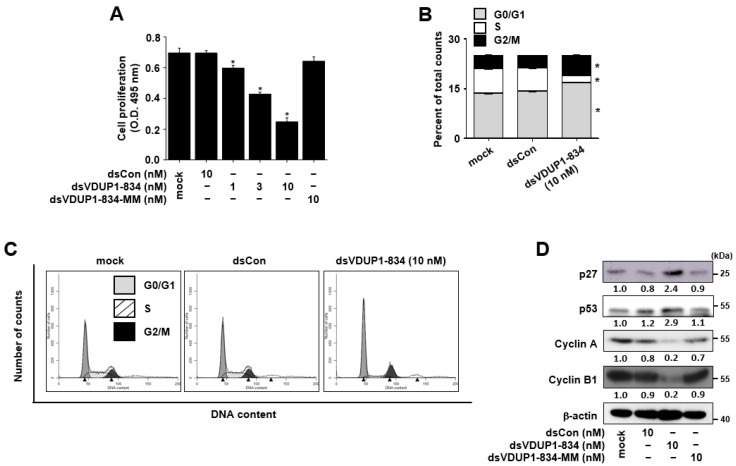
dsVDUP1-834 transfection inhibits cell growth and induces cell-cycle arrest in A549 cells. A549 cells were transfected with dsCon or indicated concentrations of saRNAs. (**A**) After 96 h, cell proliferation was measured by XTT assay. Data are represented as mean ± S.D of triplicate determinations. (**B**) Cell-cycle distribution was analyzed using flow cytometry 72 h after transfection. (**C**) A representative histogram of cell-cycle analysis. Black triangles indicate the peaks of each histogram. (**D**) Protein levels of p27, p53, cyclin A, cyclin B1, and β-actin in total cell lysates were determined by Western immunoblot analysis. Normalized relative intensities are shown below the bands. * denotes that the response is significantly different from mock-treated group as determined by Dunnett’s *t*-test at *p* < 0.05.

We then investigated the effect of dsVDUP1-834 on the cell proliferation of A549 cells by XTT assay. As shown in Figure 2A, dsVDUP1-834 transfection suppressed the proliferation of A549 cells in a concentration-dependent manner (GI_50_: 5.28 nM). We also confirmed the inhibitory effect of dsVDUP1-834 on the cell proliferation in PC-3 cells (GI_50_: 10.44 nM), in which the expression of VDUP1 gene is low, and the magnitude of VDUP1 gene induction by dsVDUP1-834 is comparable to that in A549 cells (Supplementary Figure S3). To compare the efficacy of this treatment with the treatments generally used to treat lung cancer patients, we examined the effects of doxorubicin and erlotinib in A549 cells, and the GI_50_ values of doxorubicin and erlotinib were 52 nM and 6.02 μM, respectively, in A549 cells. In contrast, dsVDUP1-834-MM transfection has no significant effect on the proliferation of A549 cells (Figure 2A).

To investigate whether VDUP1 induction by dsVDUP1-834 exerts an effect on cell-cycle distribution, the effect of dsVDUP1-834 on DNA content was analyzed by flow cytometry in A549 cells. Figure 2B,C show that the accumulation of cells in the G0/G1 and G2/M phase and the loss of cells in S phase were observed after the transfection of A549 cells with dsVDUP1-834. To further confirm, we also examined the effect of dsVDUP1-834 on the expression of cell-cycle regulators, including p27, p53, cyclin A, and cyclin B1. As shown in Figure 2D and Supplementary Figure S4, the levels of p27 and p53, inhibitors of the cell cycle, were up-regulated following dsVDUP1-834 transfection, whereas the levels of cyclin A and cyclin B1, essential components of cell-cycle progression, were dramatically down-regulated after dsVDUP1-834 transfection.

### 2.3. dsVDUP1-834 Induces DNA Demethylation and Histone Modification of VDUP1 Promoter

**Figure 3:**
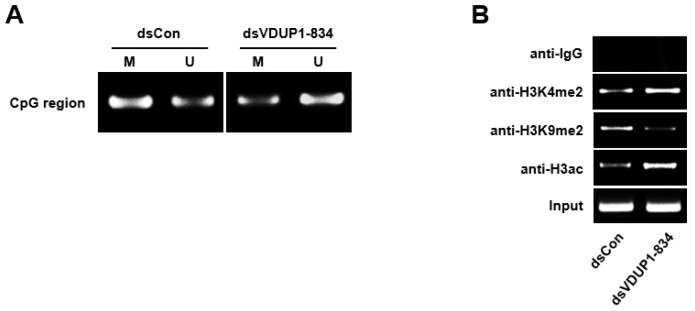
dsVDUP1-834 transfection induces DNA demethylation and histone modification. A549 cells were transfected with dsCon or dsVDUP1-834 (10 nM). (**A**) After modification with bisulfite, methylation-specific PCR was performed with primers specific to methylated (M) or unmethylated (U) CpG islands of VDUP1 promoter. (**B**) Chromatin immunoprecipitation (ChIP) assays were performed using control IgG or antibodies against H3K4me2, H3K9me2, or H3ac to pull down associated DNA. The precipitated DNA was amplified using primers specific to the dsVDUP1-834 target site, and input DNA was amplified as a loading control.

Previous reports demonstrated DNA methylation and various types of histone modifications in the promoter region following saRNA transfection [3,13,14]. To investigate the involvement of DNA methylation and histone modifications in dsVDUP1-834-induced VDUP1 up-regulation, we performed methylation-specific PCR and chromatin immunoprecipitation (ChIP) assay. As shown in Figure 3A, the VDUP1 promoter was methylated in dsCon-transfected A549 cells, whereas transfection with dsVDUP1-834 induced demethylation of the VDUP1 promoter. In addition, ChIP assay revealed that dsVDUP1-834 transfection induced dimethylation of histone 3 at lysine 4 (H3K4me2) and acetylation of histone 3 (H3ac), whereas dimethylation of histone 3 at lysine 9 (H3K9me2), a well-known marker of transcriptional repression, was decreased by dsVDUP1-834 transfection at dsVDUP1 target site in VDUP1 promoter (Figure 3B).

### 2.4. The Effects of dsVDUP1-834 on Cell Proliferation and the Expression of Cell-Cycle Regulators Were Attenuated by VDUP1 Knockdown in A549 Cells

**Figure 4:**
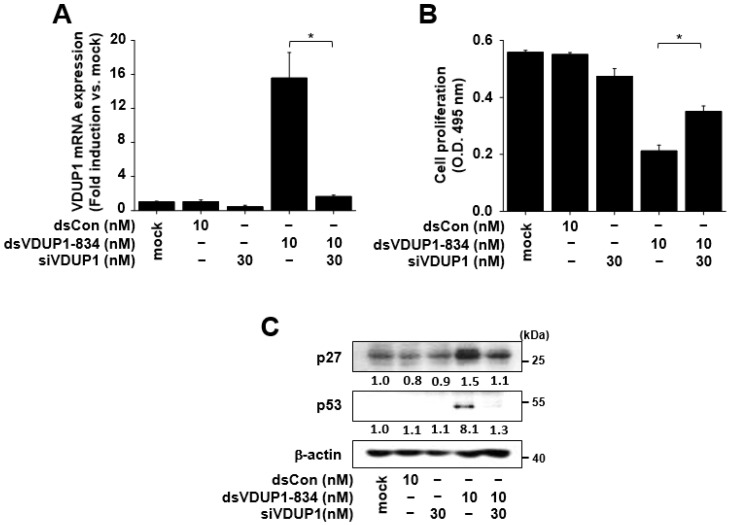
The effects of dsVDUP1-834 were attenuated by VDUP1 siRNA in A549 cells. A549 cells were transfected with dsCon, dsVDUP1-834 (10 nM) or siVDUP1 (30 nM) for 96 h. (**A**) The mRNA expression of VDUP1 was analyzed by qRT-PCR. Data are represented as mean ± S.D of triplicate determinations. (**B**) Cell proliferation was measured by XTT assay. Data are represented as mean ± S.D of triplicate determinations. (**C**) Protein levels of p27, p53, and β-actin in total cell lysates were determined by Western immunoblot analysis. Normalized relative intensities are shown below the bands. * denotes that the response is significantly different from dsVDUP1-834-treated group as determined by Dunnett’s *t*-test at *p* < 0.05.

To investigate whether the effects of dsVDUP1-834 on cell proliferation and the expression of cell-cycle regulators were mediated by induction of VDUP1 expression, we examined the effects of VDUP1 knockdown on dsVDUP1-834-mediated changes in A549 cells. As shown in Figure 4A, the increase in VDUP1 mRNA expression by dsVDUP1-834 was abolished by transfection of cells with VDUP1 siRNA. In addition, dsVDUP1-834-mediated suppression of cell proliferation was reversed by the knockdown of VDUP1 in A549 cells (Figure 4B). Further study demonstrated that dsVDUP1-834-mediated increases in the expression of p27 and p53 were also blocked by VDUP1 knockdown in A549 cells (Figure 4C and Supplementary Figure S5).

### 2.5. Induction of VDUP1 Expression by dsVDUP1-834 Is Ago2-Dependent, and Ago2 Knockdown Alleviates the Effects of dsVDUP1-834 on Cell Proliferation and the Expression of Cell-Cycle Regulators in A549 Cells

**Figure 5:**
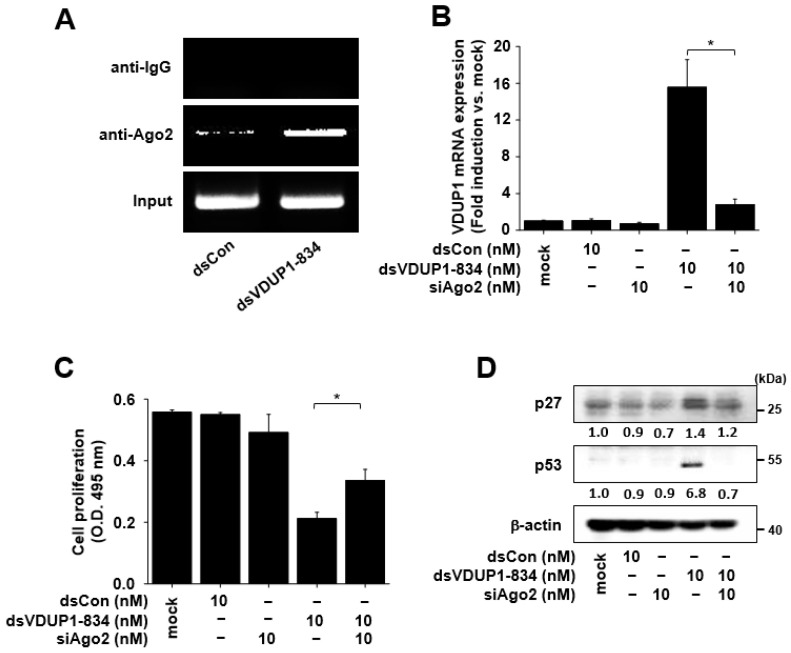
The effects of dsVDUP1-834 were attenuated by Ago2 siRNA in A549 cells. A549 cells were transfected with dsCon, dsVDUP1-834 (10 nM), or siAgo2 (10 nM) for 96 h. (**A**) ChIP assays were performed using control IgG or anti-Ago2 antibody to pull down associated DNA. The precipitated DNA was amplified by PCR using primer sets specific to dsVDUP1-834 target sites. (**B**) The mRNA expression of VDUP1 was analyzed by qRT-PCR. Data are represented as mean ± S.D of triplicate determinations. (**C**) Cell proliferation was measured by XTT assay. Data are represented as mean ± S.D of triplicate determinations. (**D**) Protein levels of p27, p53, and β-actin in total cell lysates were determined by Western immunoblot analysis. Normalized relative intensities are shown below the bands. * denotes that the response is significantly different from dsVDUP1-834-treated group as determined by Dunnett’s *t*-test at *p* < 0.05.

It is well-known that Ago2 is required for RNAa [2,4,15]. To investigate whether Ago2 is involved in VDUP1 induction by dsVDUP1-834, we performed a ChIP assay. As shown in Figure 5A, we detected an increased enrichment of Ago2 binding to the dsVDUP1-834 target site. To further investigate the requirement of Ago2 in the induction of VDUP1 gene expression by dsVDUP1-834, we transfected Ago2 siRNA (siAgo2) to knockdown Ago2 expression and then examined the effect of dsVDUP1-834 on VDUP1 mRNA expression. As shown in Figure 5B, dsVDUP1-834 induced VDUP1 mRNA expression in the absence of siAgo2. However, induction of VDUP1 mRNA expression by dsVDUP1-834 was abrogated by siAgo2 transfection.

To investigate the involvement of Ago2 in the observed effects of dsVDUP1-834, we evaluated the effect of dsVDUP1-834 on cell proliferation and the expression of cell-cycle regulators in siAgo2-transfected A549 cells. Figure 5C shows that the inhibitory effect of dsVDUP1-834 on cell proliferation was significantly reversed by the knockdown of Ago2 expression. In addition, a dsVDUP1-834-mediated increase in the levels of p27 and p53 was also reversed in siAgo2-transfected A549 cells (Figure 5D and Supplementary Figure S6).

### 2.6. dsVDUP1-834 Suppressed Tumor Growth in A549 Xenograft Model

**Figure 6:**
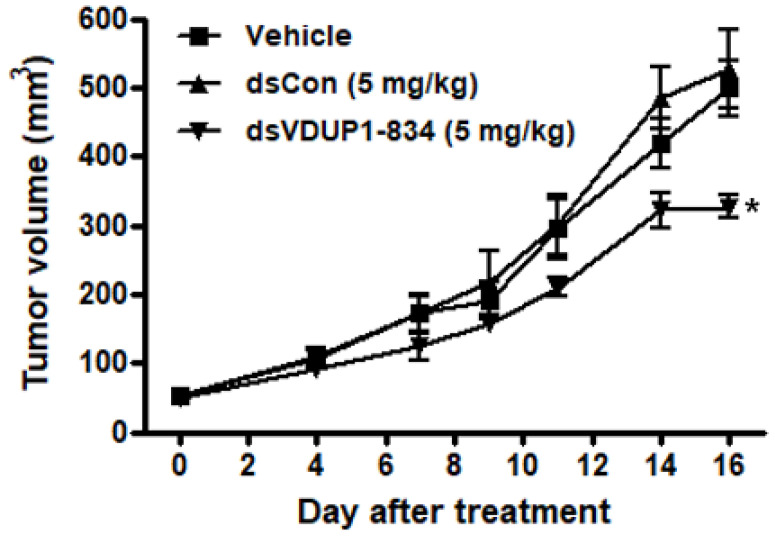
Inhibition of A549 tumor growth by dsVDUP1-834 treatment in nude mouse xenograft model. Specific pathogen-free BALB/c nude mice (*n* = 5) were implanted subcutaneously with A549 cells (9 × 10^6^ cells/mouse) and treated with vehicle, dsCon (5 mg/kg) and dsVDUP1-834 (5 mg/kg). Tumor volumes are measured, and data are represented as mean ± S.D (*n* = 5). * denotes that the response is significantly different from vehicle-treated group as determined by Dunnett’s *t*-test at *p* < 0.05.

Finally, we evaluated the anti-tumor effect of dsVDUP1-834 in the A549 xenograft model. The tumor xenograft model was established by subcutaneous implantation of A549 cells and subcutaneously implanted A549 cells formed tumors growing rapidly. The average tumor volume of the vehicle-treated group reached 499 mm^3^ on day 16 (Figure 6). However, treatment of mice with dsVDUP1-834 significantly inhibited tumor growth by 34% at day 16, whereas treatment of mice with dsCon had no significant effect on tumor growth in the A549 xenograft model (Figure 6).

## 3. Discussion

Recently, RNA-based therapeutics are gaining attention, and substantial efforts have been made toward the clinical application of RNA-based therapeutics over the past decade. Currently, 11 RNA-based therapeutics, including siRNA and anti-sense oligonucleotide, are approved by the FDA (Food and Drug Administration) and/or the EMA (European Medicines Agency) [16]. However, only antagonism of a target gene is possible with siRNA or anti-sense oligonucleotide, and therefore, diseases caused by inactivation of reduced expression of genes cannot be corrected by these modalities [17]. On the other hand, RNAa can induce inactivated or reduced expression of genes. In the present study, we demonstrated that dsVDUP1-834 induces VDUP1 gene expression and inhibits cell proliferation in A549 lung cancer cells. We also showed that dsVDUP1-834 induces cell-cycle arrest by modulating cell-cycle regulators, including p27, p53, cyclin A, and cyclin B1. In addition, the results presented in this report showed that epigenetic modifications of the promoter region induced by dsVDUP1-834 are similar to those by saRNA, suggesting that dsVDUP1-834 might induce VDUP1 gene expression via RNAa mechanism. Collectively, these results suggest that dsVDUP1-834 might be a therapeutic candidate for the treatment of cancers related to VDUP1 down-regulation.

It has been reported that VDUP1 induces cell-cycle arrest and apoptosis, regulates mitochondrial function, and inhibits growth and metastasis [18]. p27 and p53 are well-known cell-cycle regulators. p27 binds and inhibits cyclin A- or cyclin E-associated cyclin-dependent kinase 2 activity [19]. p53 is known as an important regulator of G2/M transition, and it induces cell-cycle arrest by inhibition of cyclin-dependent kinase 1 and repression of the cyclin B1 gene expression [20]. VDUP1 is known to increase the stability of p27, a cyclin-dependent kinase inhibitor, by inhibiting JAB1 [21]. In this study, we demonstrated that dsVDUP1-834 up-regulates p27 expression in A549 cells, and this was abrogated by knockdown of VDUP1 gene expression, suggesting that the induction of p27 expression by dsVDUP1-834 might be mediated by induction of VDUP1 expression. In consistent with our results, D-allose, a rare sugar, inhibited cell growth via induction of VDUP1 and subsequent cell-cycle arrest mediated by up-regulation of p27 in cancer cells [22,23]. VDUP1 is also implicated in the regulation of p53. Suh et al. reported that VDUP1 induced p53 stabilization via inhibition of MDM2-mediated p53 ubiquitination in cancer cells. Our results also showed that the expression of p53 is increased by dsVDUP1-834 transfection and VDUP1 knockdown reversed dsVDUP1-834-mediated up-regulation of p53 expression, suggesting that VDUP1 expression is necessary for the dsVDUP1-mediated increase in p53 expression. In addition, it has been reported that cyclin A is a downstream target gene of VDUP1, which can directly act as a transcriptional repressor of cyclin A gene expression [24]. These results indicate that the effects of dsVDUP1-834 on cell proliferation might be mediated, at least in part, by modulating cell-cycle regulators, such as p27 and p53.

It has been reported that RNAa induces epigenetic changes by histone modification, such as the gain of active marks, including H3K3me2 and H3K4me3, and the loss of repressive marks, including H3K9me and H3K28me3 [2,3,13,25,26]. In this report, we confirmed that dsVDUP1-834 transfection induces epigenetic changes involving an increased H3K4me2 and a decreased H3K9me2 at the target site of the VDUP1 promoter. We also demonstrated that dsVDUP1-834 increases the acetylation of H3, another marker of promoter activation. These results suggest that histone modification might be involved in dsVDUP1-834-mediated induction of VDUP1 gene expression. It is also well-known that DNA hypermethylation is an important mechanism for the inactivation of tumor suppressor genes [27]. In this report, we showed that the promoter region of VDUP1 was demethylated by dsVDUP1-834 transfection in A549 cells. Collectively, these results suggest that dsVDUP1-834-mediated up-regulation of VDUP1 gene expression might be mediated by epigenetic changes, including both histone modification and promoter demethylation.

The Argonaute protein family has been known to play an important role in RNA silencing processes as a component of the RNA-induced silencing complex (RISC) [28]. Moreover, Li et al. reported that the Argonaute proteins, especially Ago2, are required for RNAa [2]. Further studies demonstrated that Ago2 is required for processing saRNAs such as siRNA maturation, and the endonuclease activity of Ago2 cleaves and discards the passenger stand to form an active RNA-Ago complex capable of recognizing complementary sequences [29,30]. In this report, we showed that dsVDUP1-834-mediated induction of VDUP1 expression is abrogated by Ago2 knockdown, and this resulted in a reversal of dsVDUP1-834-mediated inhibition of cell proliferation and modulation of cell-cycle regulators. From these results, it is assumed that Ago2 is required for the induction of the VDUP1 gene expression by dsVDUP1-834.

Taken together, we demonstrated that dsVDUP1-834 induces VDUP1 gene expression and inhibits cell proliferation in A549 cells. Our results also show that dsVDUP1-834 induced up-regulation of p27 and p53, which might contribute to cell growth inhibition. We also demonstrated that dsVDUP1-834 induces DNA demethylation and histone modification and requires Ago2 for its effect. Collectively, the results presented in this report indicate that VDUP1 might be induced by RNAa and suggest the potential therapeutic use of dsVDUP1-834 for cancer treatment by targeted activation of VDUP1.

## 4. Materials and Methods

### 4.1. Preparation of dsRNAs

dsRNAs were designed by previously detailed general design rules [31,32] and chemically synthesized by Bioneer (Daejeon, Korea). dsVDUP1-834 was designed to target the VDUP1 promoter at positions -834 relative to the transcription start site, and dsVDUP1-834-MM was designed by changing the last five bases (relative to the 5′ end of the anti-sense strand) of dsVDUP1-834 (Figure 1A). A control saRNA (dsCon) lacking homology to any human sequences was also designed as described previously [2]. A BLAST search (http://blast.ncbi.nlm.nih.gov/Blast/cgi, accessed on 22 March 2013) was performed to exclude the possibility that these dsRNAs target other human genes. All dsRNA sequences are listed in Supplementary Table S1.

### 4.2. Cell Culture and Transfection

Human lung cancer A549 (CCL-185^TM^) cells were purchased from American Type Culture Collection (ATCC, Rockville, MD, USA) and cultured in RPMI 1640 Medium (Gibco, Grand Island, NY, USA) supplemented with 10% fetal bovine serum, penicillin (100 U/mL) and streptomycin (100 mg/mL). Cells were maintained at 37 °C in a humidified atmosphere containing 5% CO_2_. dsRNAs were transfected using Lipofectamine RNAiMax reagent (Invitrogen, Carlsbad, CA, USA) according to the manufacturer’s instructions.

### 4.3. RNA Isolation and Quantification of mRNA Expression

Total cellular RNA was extracted using RNeasy Plus Mini Kit (Qiagen, Valencia, CA, USA) with RNase-Free DNase Set (Qiagen) according to the manufacturer’s instructions. cDNA was generated from total RNA by reverse transcription using AccuPower RT PreMix (Bioneer). The resulted cDNA was amplified by qPCR in conjunction with Power SYBR Green PCR Master Mix (Invitrogen, Carlsbad, CA, USA) or by regular RT-PCR. For qPCR, samples were amplified by 45 cycles of denaturation (95 °C for 15 s) and amplification (60 °C for 1 min using ABI 7500 Sequence Detection System (Applied Biosciences, Foster City, CA, USA). The relative gene expression levels relative to the control gene (β-actin) was calculated by the 2^−∆∆Ct^ method. For regular RT-PCR, the amplification program consisted of an initial denaturation step (95 °C for 3 min), 33 cycles of denaturation (95 °C for 30 s), annealing (58 °C for 30 s), and extension (72 °C for 60 s) followed by a final extension at 72 °C for 5 min. All primer sequences are listed in Supplementary Table S1.

### 4.4. Cell Proliferation Assay

Cell proliferation assays were performed using a Cell Proliferation Kit II (Roche Applied Science, Mannheim, Germany) according to the manufacturer’s instructions. Briefly, the XTT labeling mixture was prepared by mixing 50 volumes of 1 mg/mL sodium 3′-[1-(phenylaminocarbonyl)-3,4-tetrazolium]-bis(4-methoxy-6-nitro)benzene sulfonic acid hydrate with 1 volume of 0.383 mg/mL of N-methyldibenzopyrazine methyl sulfate, added to the cultures and incubated for 2 h at 37 °C. Absorbance was measured at 495 nm with a reference wavelength of 650 nm.

### 4.5. Western Immunoblot Analysis

Total protein extracts were prepared by lysing cells in Cell Lysis Buffer (Cell Signaling Technology, Beverly, MA, USA) with protease inhibitor cocktail (Merck Millipore, Billerica, MA, USA) and phosphatase inhibitors (Sigma-Aldrich, St. Louis, MO, USA). Protein concentrations in the lysates were determined using a BCA Protein Assay Kit (Pierce Biotechnology, Waltham, MA, USA) according to the manufacturer’s instructions. Protein extracts were separated by 8~12% sodium dodecyl sulfate-polyacrylamide gel electrophoresis and transferred to nitrocellulose membranes. The membranes were incubated with blocking buffer (Tris-buffered saline containing 0.05% Tween 20 and 5% non-fat dried milk) and probed with the primary antibodies against VDUP1 (MBA International, Woburn, MA, USA), p27, p53, cyclin A, cyclin B1, or β-actin (Cell Signaling Technology). After washing, membranes were probes with horseradish peroxidase (HRP)-conjugated secondary antibodies and visualized using an Immobilon Western Chemiluminescent HRP substrate (Merck Millipore). The band intensities are quantified by ImageJ 1.53a and normalized by that of β-actin.

### 4.6. Methylation Analysis

Genomic DNA was isolated using QIAamp DNA Mini Kit (Qiagen) according to the manufacturer’s instructions. Bisulfite conversion of DNA (500 ng/reaction) was performed using EZ DNA Methylation Kit (Zymo Research, Irvine, CA, USA). Methylated and unmethylated DNAs were amplified in separate reactions with primers designed using the MethPrimer program, and primer sequences used for this analysis are listed in Supplementary Table S1. Methylation-specific PCR reactions were performed using the EX TaqDNA polymerase Hot-Start version (Takara, Shiga, Japan) according to the manufacturer’s instructions.

### 4.7. Chromatin Immunoprecipitation (ChIP) Assay

ChIP assay was performed as described previously with slight modifications [33]. Briefly, A549 cells were cross-linked with 1% formaldehyde for 10 min at room temperature. The cross-linking reaction was stopped by adding glycine stock solution to a final concentration of 0.125 M. DNA was sheared to an average size of ~500 bp using a Vibra cell sonicator (Sonics and Materials Inc., Newtown, CT, USA). The sonicated mixture was centrifuged at 14,000 rpm for 15 min at 4 °C, and the supernatant was collected. Immunoprecipitation was performed using 5 μg/sample of normal mouse IgG (Santa Cruz Biotechnology, Dallas, TX, USA), anti-Ago2 (Abcam, Cambridge, MA, USA), anti-H3K4me2, anti-H3K9me2, or H3ac (Cell Signaling Technology). The samples were incubated with Protein A/G Agarose suspension (Merck Millipore) for 2 h at 4 °C and sequentially washed with low salt, high salt, and TE buffer. Elutes were collected and reverse cross-linked at 65 °C overnight. Samples were subsequently treated with RNase A and Proteinase K followed by phenol/chloroform extraction and analyzed by PCR. PCR primers used for ChIP analysis are listed in Supplementary Table S1.

### 4.8. Cell-Cycle Analysis

Cell-cycle distributions were analyzed as described previously [14]. Cells were harvested and centrifuged at 1000× *g* for 5 min and washed with ice-cold PBS. The cells were fixed in 70% ethanol overnight at 4 °C, stained in 1 mL of Krishan Buffer (0.1% sodium citrate, 0.03% Triton X-100, 0.02 mg/mL RNase A, 0.05 mg/mL propidium iodide) for 1 h at 4 °C and analyzed using a FACSCalibur flow cytometer (BD Biosciences, San Jose, CA, USA). The data were analyzed using the ModFit LT program (Verity Software House, Maine, ME, USA).

### 4.9. Human Tumor Xenograft Model

Female BALB/c nude mice (5 weeks old) were purchased from Charles River Japan (Yokohama, Japan) and were housed under specific pathogen-free conditions. Rooms are maintained under a 12 h light-dark cycle at 21 ± 2 °C. Animals were allowed to acclimate to the local environment for 1 week before use. All animal experiments were approved by the Institutional Animal Care and Use Committee of the Korea Research Institute of Bioscience and Biotechnology (Approval #: KRIBB-AEC-14175). A549 cells were collected and suspended in a serum-free media. Cell suspensions (5 × 10^6^ cells) were injected subcutaneously into BALB/c nude mice. When tumor volumes reached about 50 mm^3^, mice were randomly divided into three groups. Then, 65 μL of dsCon and dsVDUP1-834 dissolved in Opti-MEM was mixed with 35 μL of Lipofectamine RNAiMax before administration. Vehicle, dsCon (5 mg/kg) or dsVDUP1-834 (5 mg/kg) were administered intraperitoneally (3 times a week for 2 weeks). Tumor volumes were measured on days 0, 4, 7, 9, 11, 14, and 16 using Vernier calipers and calculated by the formula: length (mm) × width (mm) × height (mm)/2.

### 4.10. Statistical Analysis

Results are expressed as the mean SD. One-way ANOVA followed by Dunnett’s *t*-test was used for statistical analysis using GraphPad Prism (GraphPad Software, La Jolla, CA, USA). The criteria for statistical significance were set at * *p* < 0.05.

## Supplementary Materials

The following supporting information can be downloaded at: https://www.mdpi.com/article/10.3390/ijms23147743/s1.
