# Supplementary material for: Targeted Induction of Endogenous VDUP1 by Small Activating RNA Inhibits the Growth of Lung Cancer Cells
Source: Int J Mol Sci. 2022 Jul 13;23(14):7743. doi: 10.3390/ijms23147743 (PMC9323751; doi:10.3390/ijms23147743)
Supplement: Supplementary file 1 [file ijms-23-07743-s001.zip › ijms-1785125-supplementary.pptx]

## Slide 1
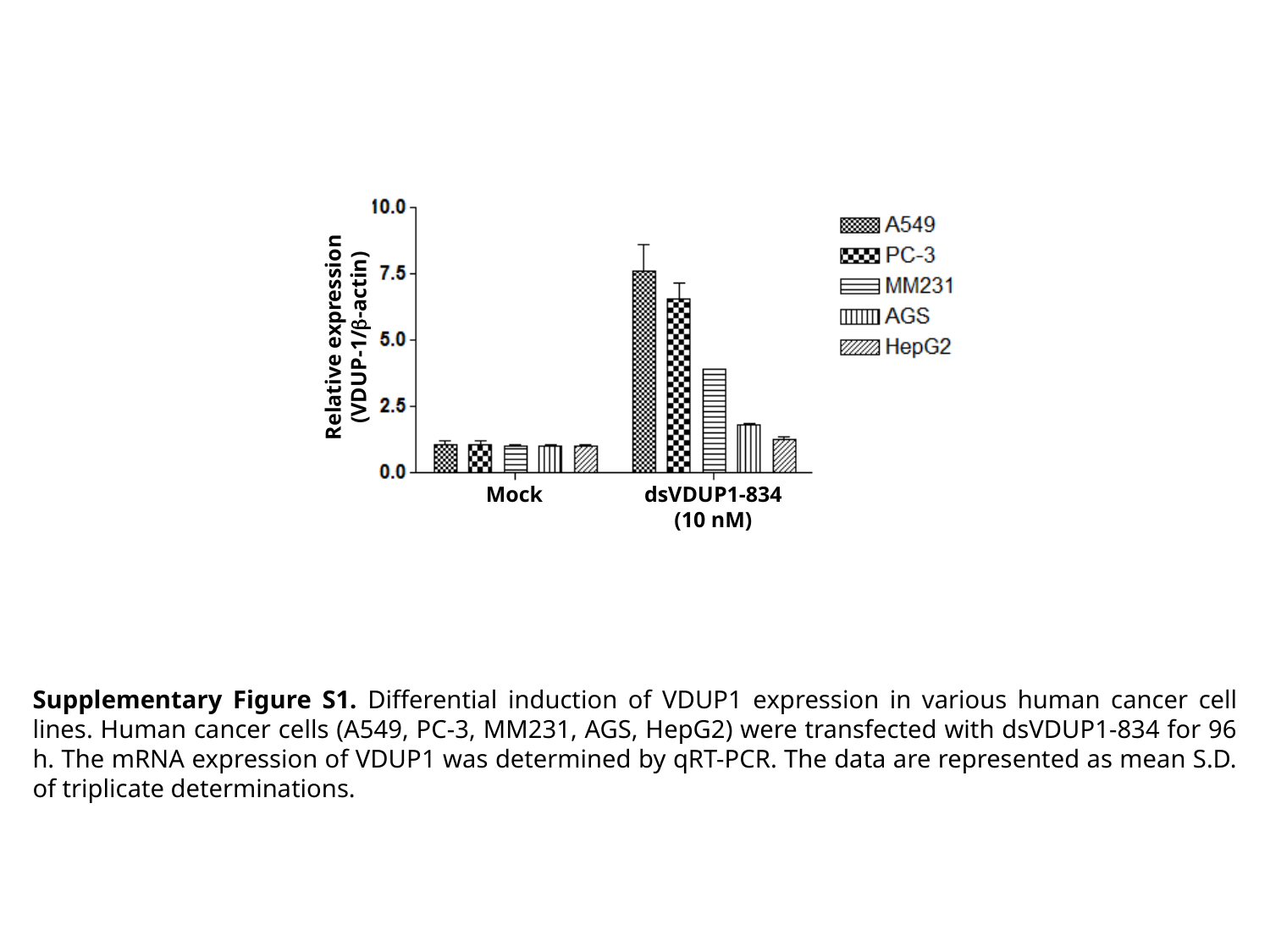

Relative expression
(VDUP-1/b-actin)
Mock
dsVDUP1-834
(10 nM)

## Slide 2
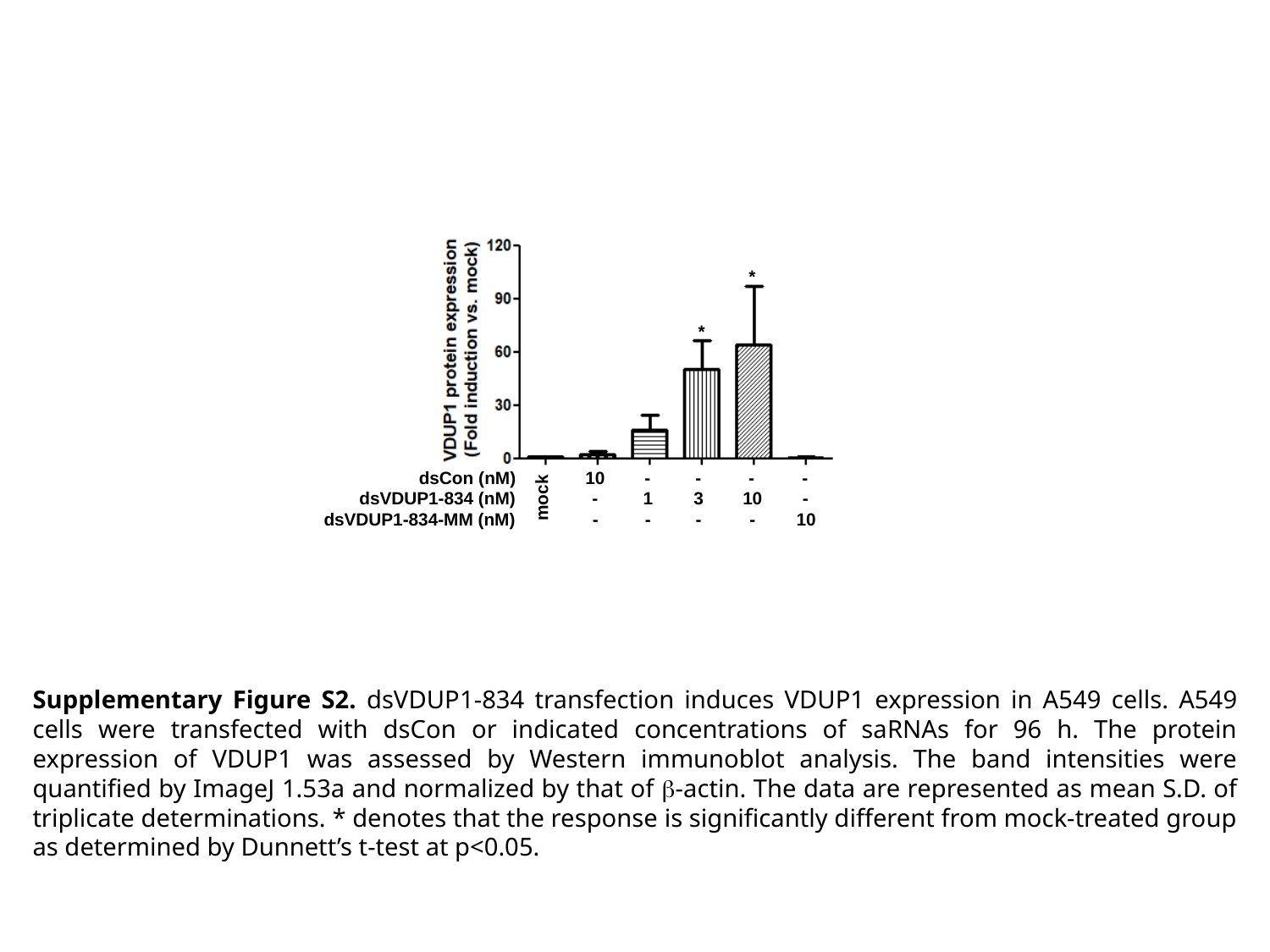

*
*
dsCon (nM)
10
-
-
-
-
mock
dsVDUP1-834 (nM)
-
1
3
10
-
dsVDUP1-834-MM (nM)
-
-
-
-
10

## Slide 3
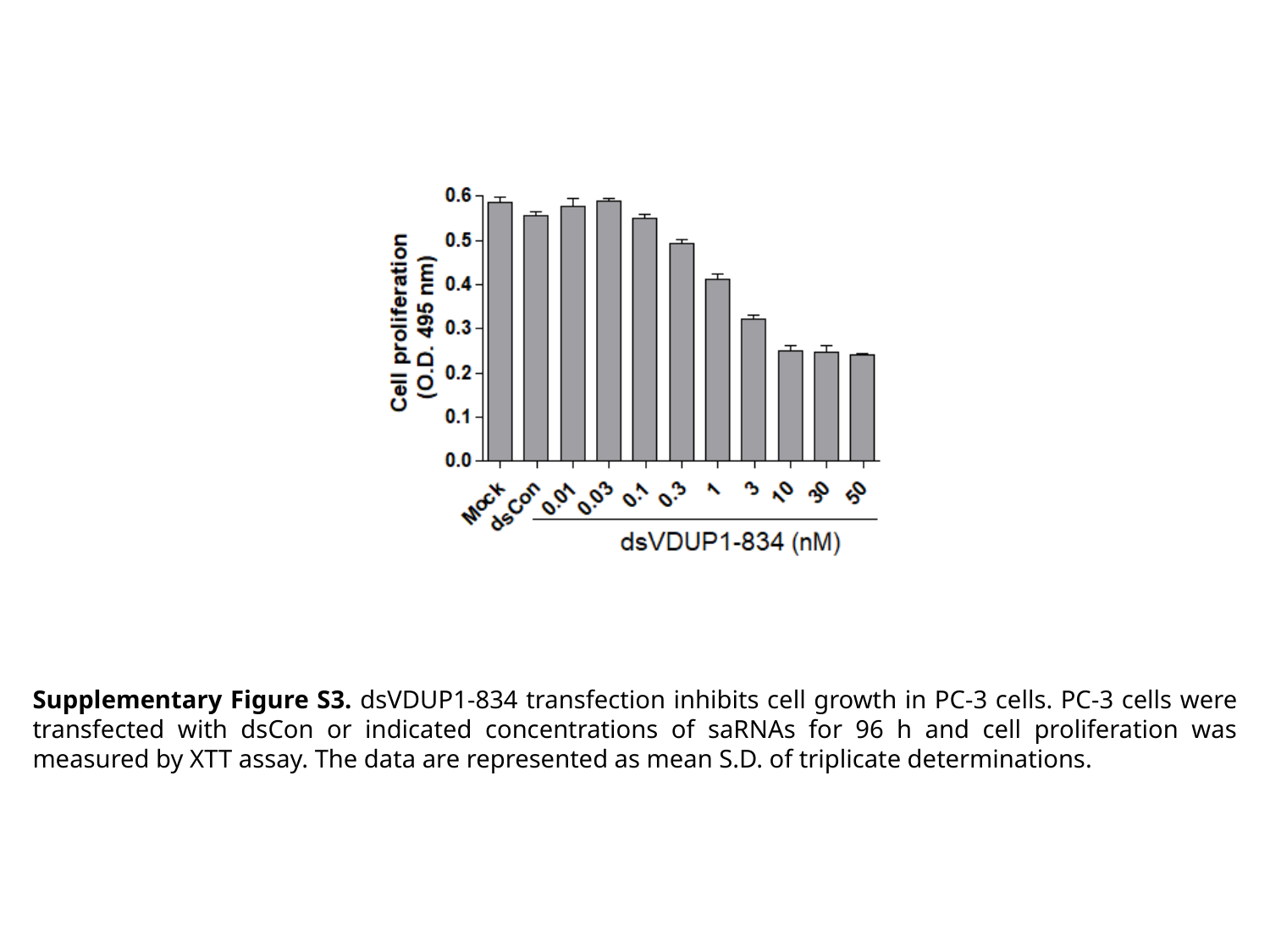

## Slide 4
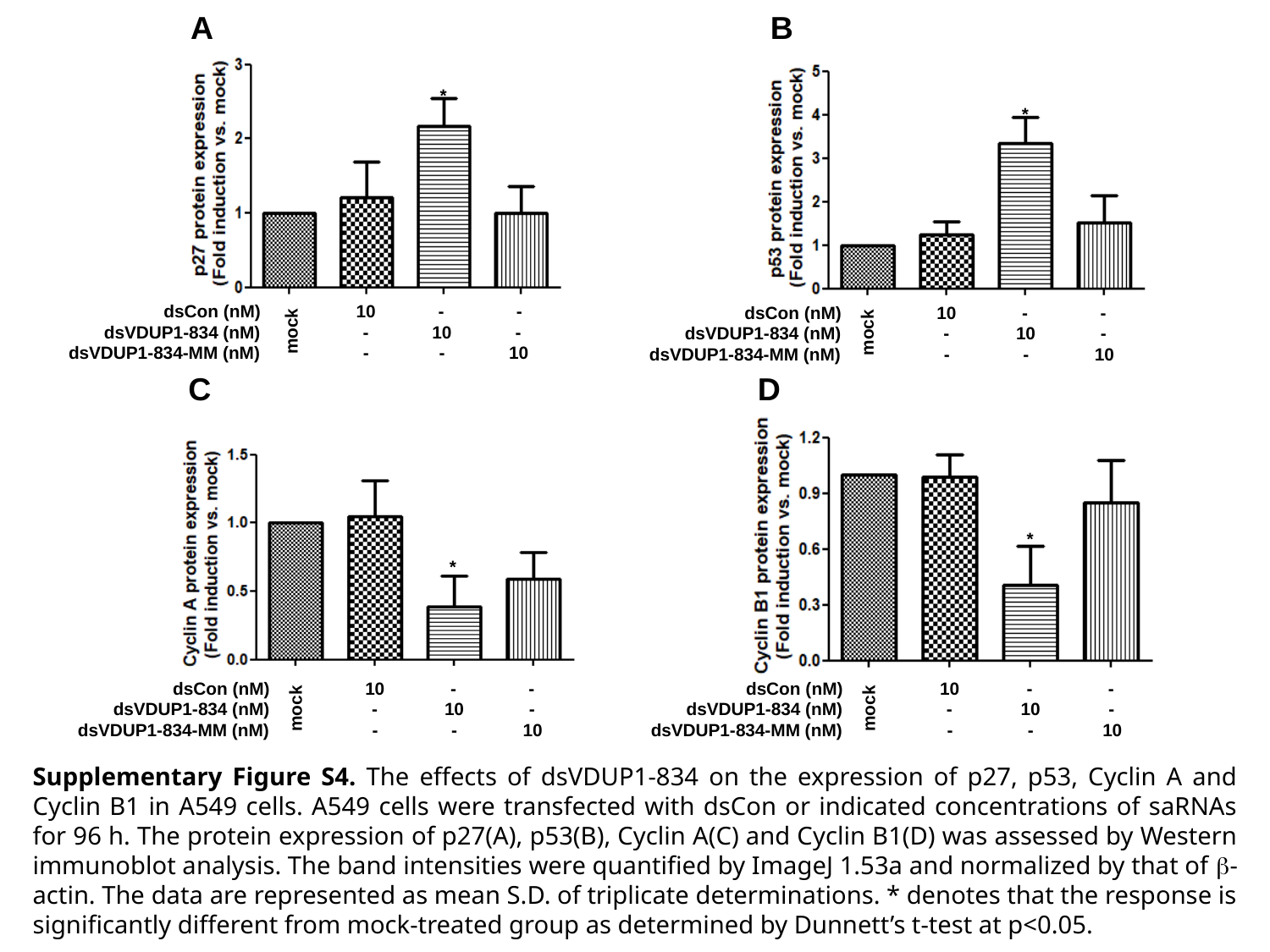

A
B
*
*
dsCon (nM)
10
-
-
dsCon (nM)
10
-
-
mock
dsVDUP1-834 (nM)
-
10
-
mock
dsVDUP1-834 (nM)
-
10
-
dsVDUP1-834-MM (nM)
-
-
10
dsVDUP1-834-MM (nM)
-
-
10
D
C
*
*
dsCon (nM)
10
-
-
dsCon (nM)
10
-
-
mock
mock
dsVDUP1-834 (nM)
-
10
-
dsVDUP1-834 (nM)
-
10
-
dsVDUP1-834-MM (nM)
-
-
10
dsVDUP1-834-MM (nM)
-
-
10

## Slide 5
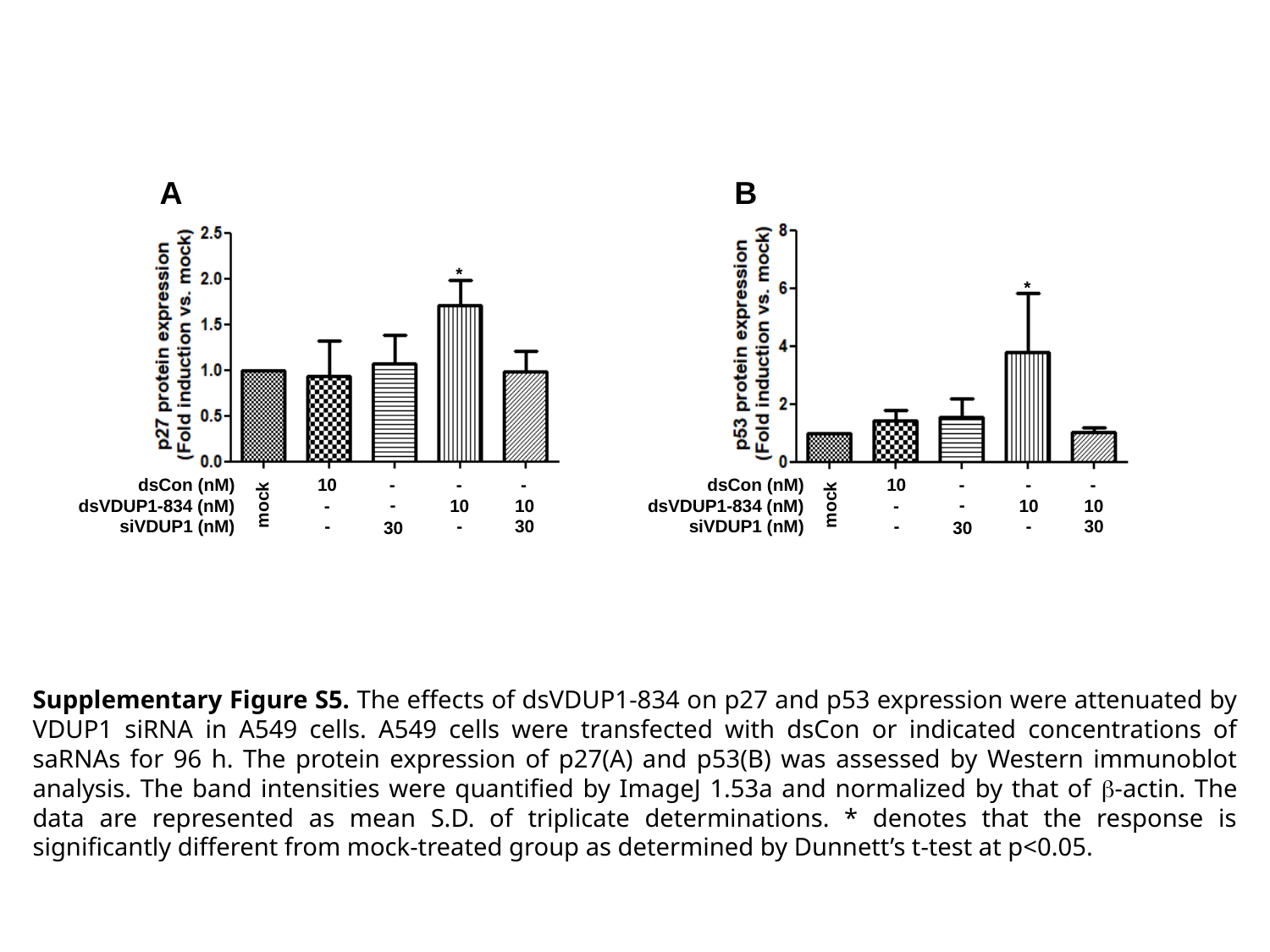

A
B
*
*
dsCon (nM)
10
-
-
-
dsCon (nM)
10
-
-
-
-
-
mock
mock
dsVDUP1-834 (nM)
-
10
10
dsVDUP1-834 (nM)
-
10
10
siVDUP1 (nM)
-
-
30
siVDUP1 (nM)
-
-
30
30
30

## Slide 6
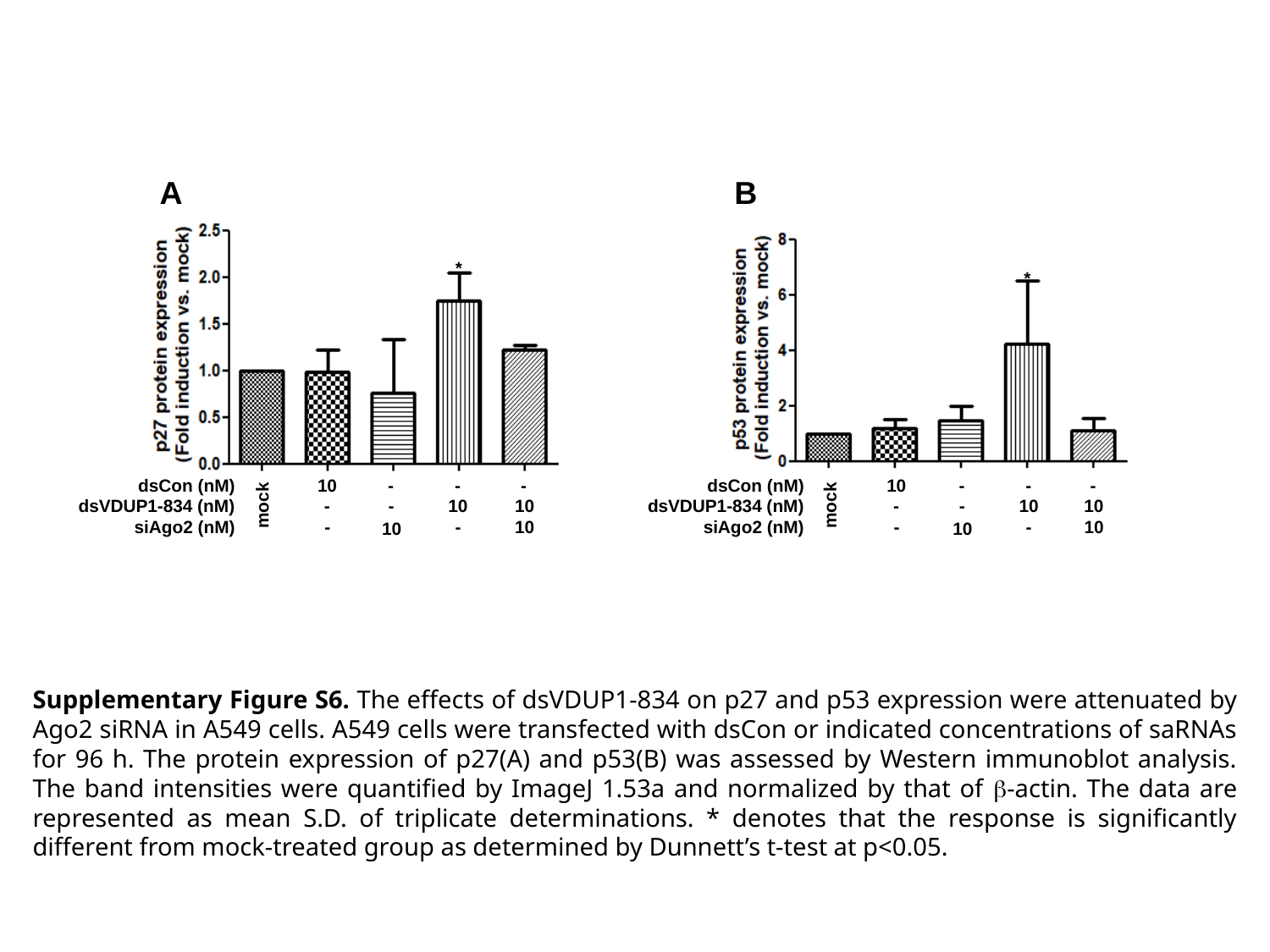

A
B
*
*
dsCon (nM)
10
-
-
-
dsCon (nM)
10
-
-
-
-
-
mock
mock
dsVDUP1-834 (nM)
-
10
10
dsVDUP1-834 (nM)
-
10
10
siAgo2 (nM)
-
-
10
siAgo2 (nM)
-
-
10
10
10
